# The ADC API: A Web API for the Programmatic Query of the AIRR Data Commons

**DOI:** 10.3389/fdata.2020.00022

**Published:** 2020-06-17

**Authors:** Scott Christley, Ademar Aguiar, George Blanck, Felix Breden, Syed Ahmad Chan Bukhari, Christian E. Busse, Jerome Jaglale, Srilakshmy L. Harikrishnan, Uri Laserson, Bjoern Peters, Artur Rocha, Chaim A. Schramm, Sarah Taylor, Jason Anthony Vander Heiden, Bojan Zimonja, Corey T. Watson, Brian Corrie, Lindsay G. Cowell

**Affiliations:** ^1^Department of Population and Data Sciences, UT Southwestern Medical Center, Dallas, TX, United States; ^2^Centre for Information Systems and Computer Graphics, Institute for Systems and Computer Engineering, Technology and Science, Porto, Portugal; ^3^Department of Informatics Engineering, Faculty of Engineering, University of Porto, Porto, Portugal; ^4^Department of Molecular Medicine, Morsani College of Medicine, University of South Florida, Tampa, FL, United States; ^5^Department of Biological Sciences, Simon Fraser University, Burnaby, BC, Canada; ^6^Division of Computer Science, Mathematics and Science (Healthcare Informatics), College of Professional Studies, St. John's University, New York, NY, United States; ^7^Division of B Cell Immunology, German Cancer Research Center (DKFZ), Heidelberg, Germany; ^8^Department of Genetics and Genome Sciences, Precision Immunology Institute, Icahn School of Medicine at Mount Sinai, New York, NY, United States; ^9^Division of Vaccine Discover, La Jolla Institute for Immunology, La Jolla, CA, United States; ^10^Department of Medicine, University of California, San Diego, San Diego, CA, United States; ^11^Vaccine Research Center, National Institute of Allergy and Infectious Diseases, NIH, Bethesda, MD, United States; ^12^10x Genomics, Pleasanton, CA, United States; ^13^Department of Bioinformatics and Computational Biology, Genentech Inc., South San Francisco, CA, United States; ^14^Department of Biochemistry and Molecular Genetics, University of Louisville School of Medicine, Louisville, KY, United States

**Keywords:** community standards, data sharing, immunology, Rep-Seq, repertoire analysis, antibody, immunoglobulin

## Abstract

The Adaptive Immune Receptor Repertoire (AIRR) Community is a research-driven group that is establishing a clear set of community-accepted data and metadata standards; standards-based reference implementation tools; and policies and practices for infrastructure to support the deposit, curation, storage, and use of high-throughput sequencing data from B-cell and T-cell receptor repertoires (AIRR-seq data). The AIRR Data Commons is a distributed system of data repositories that utilizes a common data model, a common query language, and common interoperability formats for storage, query, and downloading of AIRR-seq data. Here is described the principal technical standards for the AIRR Data Commons consisting of the AIRR Data Model for repertoires and rearrangements, the AIRR Data Commons (ADC) API for programmatic query of data repositories, a reference implementation for ADC API services, and tools for querying and validating data repositories that support the ADC API. AIRR-seq data repositories can become part of the AIRR Data Commons by implementing the data model and API. The AIRR Data Commons allows AIRR-seq data to be reused for novel analyses and empowers researchers to discover new biological insights about the adaptive immune system.

## Introduction

The Adaptive Immune Receptor Repertoire (AIRR) Community is a research-driven group that is organizing and coordinating stakeholders in the use of next-generation sequencing technologies to study antibody/B-cell and T-cell receptor repertoires (Breden et al., 2017). The use of high-throughput sequencing for profiling B-cell and T-cell receptors has resulted in a rapid increase in data generation. It is timely, therefore, for the AIRR community to establish a clear set of community-accepted data and metadata standards; standards-based reference implementation tools; and policies and practices for infrastructure to support data deposit, curation, storage, and use. These actions are in accordance with the internationally accepted policies of numerous funding agencies and publishers to make scientific data findable, accessible, interoperable and reusable (FAIR) (Wilkinson et al., 2016). Since its formation in 2015, the AIRR Community has defined minimal experimental information (MiAIRR) for describing published AIRR-seq datasets (Rubelt et al., 2017) and schema and file formats for annotated rearrangement data (Vander Heiden et al., 2018). The AIRR Common Repository Working Group (CRWG) was tasked to develop a set of recommendations that promote the deposit, sharing, and use of AIRR-seq data. These recommendations were developed and refined following community discussions at the AIRR Community Meetings in 2016 and 2017 and were approved through a vote by the AIRR Community at the AIRR Community Meeting in December 2017. Subsequent revisions for the current v0.6.0 recommendations were ratified at the AIRR Community Meeting in May 2019. The recommendations (1) state the general principles for sharing of AIRR-seq data; (2) outline the characteristics of compliant repositories for data deposit, storage, and access; and (3) describe a distributed system of compliant repositories for AIRR-seq data, linked by a central registry, formally called the AIRR Data Commons. The AIRR Data Commons is consistent with an intermediate distributed model (Contreras and Reichman, 2015) that is comprised of a system of multiple, distributed data repositories, is supported by a central registry, and utilizes a common data model and interoperability formats. Here is described the principal technical standards for the AIRR Data Commons consisting of the AIRR Data Model for repertoires and rearrangements, the AIRR Data Commons (ADC) API for programmatic query of data repositories, a reference implementation for ADC API services, and tools for querying and validating data repositories that support the ADC API. The definition of the AIRR Data Model and ADC API provides the mechanism for investigators establishing their own AIRR-seq data repositories to integrate those repositories into the AIRR Data Commons.

**Figure 1:**
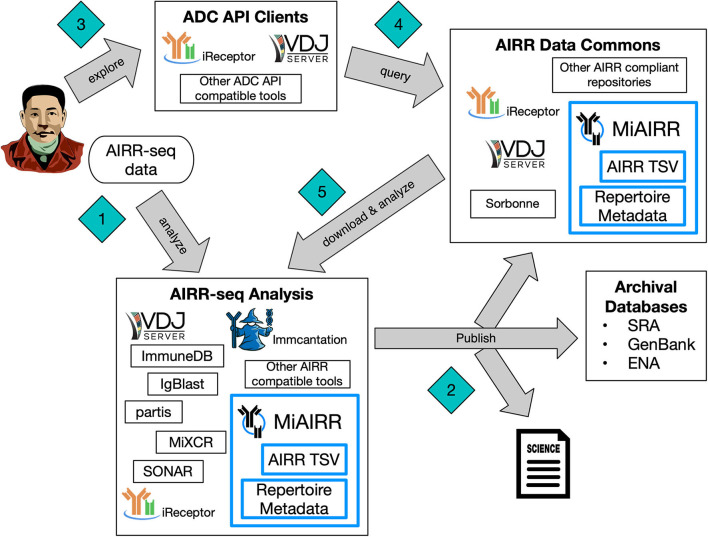
AIRR Standards Ecosystem. The AIRR Standards consisting of MiAIRR, the AIRR Data Model with the repertoire metadata schema and the AIRR TSV for rearrangements, and the ADC API, creates a cohesive ecosystem making AIRR-seq data findable, accessible, interoperable, and reusable. A typical entrance (Diamond #1) is when a researcher generates their own AIRR-seq data and performs analysis on it. The AIRR Standards allows the researcher to utilize a diverse set of analysis tools by providing interoperability of the data between the tools. Furthermore, when the researcher publishes (Diamond #2) their findings, the AIRR Standards facilitates conformance to data reporting and sharing by international funder and journal policies, archival of the raw sequencing data into databases of the International Nucleotide Sequence Database Collaboration, and storage of metadata and annotations in the AIRR Data Commons. Another starting point (Diamond #3) is when a researcher explores the AIRR Data Commons using a variety of web interfaces and tools that can communicate through the ADC API, and these ADC API clients query (Diamond #4) the AIRR Data Commons on behalf of the researcher. The AIRR Standards ensures reusability of the data downloaded (Diamond #5) from the AIRR Data Commons with the same, diverse set of analysis tools. As the AIRR Data Commons grows, researchers can utilize comparative analyses between their own data (Diamond #1) and data from the AIRR Data Commons (Diamond #5) to provide novel biological insights into the adaptive immune system.

Figure 1 illustrates how the AIRR Standards creates an ecosystem for researchers that makes AIRR-seq data FAIR (Wilkinson et al., 2016). Researchers interact with this ecosystem through analysis of their own AIRR-seq data, exploration and query of the AIRR Data Commons, or a combination of the two. The AIRR Data Model, described in more detail in Section AIRR Data Model, provides interoperability between a diverse set of analysis tools, such as IgBlast (Ye et al., 2013), Immcantation suite (Gupta et al., 2015), ImmuneDB (Rosenfeld et al., 2017), iReceptor (Corrie et al., 2018), MiXCR (Bolotin et al., 2015), partis (Ralph and Matsen, 2016), SONAR (Schramm et al., 2016), and VDJServer (Christley et al., 2018). A complete list of tools that support the AIRR Standards can be found at the documentation website (https://docs.airr-community.org). Researchers can explore the AIRR Data Commons with graphical user interfaces, such as those provided by VDJServer and iReceptor, that hides the complexity of the distributed system of multiple data repositories. Alternatively, researchers can directly query the data repositories themselves. The ADC API, described in Section AIRR Data Commons (ADC) API, provides a common query language and data exchange format to ensure interoperability among all of the data repositories. Furthermore, the AIRR Standards used pervasively throughout the whole ecosystem allows AIRR-seq data to be reused for novel analyses and empowers researchers to discover new biological insights about the adaptive immune system.

## AIRR Data Model

The MiAIRR data standard defines the minimal information required for interpretation and comparison of AIRR-seq datasets (Rubelt et al., 2017). The standard defines a set of data elements for this information and organizes them into six high-level sets.

- Study, Subject, and Diagnosis.
- Sample Collection.
- Sample Processing and Sequencing.
- Raw Sequences.
- Data Processing.
- Processed Sequences with Annotations.

However, beyond these sets, MiAIRR does not define any structure, data model, or relationship between the data elements. This provides flexibility for the information to be stored in various data repositories but does not ensure the interoperability and reusability of that information when consumed by computer programs. The AIRR Data Model overcomes these issues by defining a schema for the MiAIRR data elements, structuring them within schema objects, defining the relationships between those objects, and defining a file format. The schema for the AIRR Data Model is provided in a machine-readable YAML document that follows the OpenAPI v2.0 specification and corresponds to v1.3.0 of the AIRR Schema. It is a significant extension of the AIRR Schema v1.2.0 that provided a data representation standard for reference alignments and rearrangements annotations (Vander Heiden et al., 2018). The primary schema objects of the AIRR Data Model are summarized in Table 1.

**Table 1:** Primary schema objects of the AIRR data model.

**Schema object**	**Description**
Study	Information about the experimental study design, including the title of the study, laboratory contact information, funding, and linked publications.
Subject	Information about the study cohorts and individual subjects, including species, sex, age, and ancestry.
Diagnosis	Information about disease state(s), therapies, and study group membership (e.g., control versus disease).
Sample	Information about the origin and expected composition of the biological sample(s). This set aims to capture essential information about the collection of a sample, including its source (e.g., anatomical site), its provenance (provider), and the experimental condition (e.g., the time point during the course of a disease or treatment).
CellProcessing	Information about the cell subset being profiled, as defined by the investigator, and the flow cytometry or other markers used to select the subset. Additional information includes the number of cells per sample and whether cells were prepared in bulk or captured as single cells.
NucleicAcidProcessing	Information about nucleic acid sample type (e.g., RNA versus DNA) and how immune-receptor gene rearrangements were amplified and sequenced (for example, RACE-PCR versus multiplex PCR, paired PCR, and/or varying read length and sequencing chemistries).
SequencingRun	Information about the sequencing run, such as the number of reads, read lengths, quality control parameters, the sequencing kit and instrument(s) used, and run batch number. Also includes information about the raw data for the sequencing run (e.g., FASTQ files).
DataProcessing	Information about the data processing to transform the raw sequencing data into **Rearrangements**.
Repertoire	Composite object that combines the schema objects **Study**, **Subject**, **Diagnosis**, **Sample**, **CellProcessing**, **NucleicAcidProcessing**, **SequencingRun**, and **DataProcessing**. Each **Repertoire** has a unique identifier *repertoire_id* for linking with other data files, e.g., **Rearrangements**.
Rearrangements	Annotated sequences describing adaptive immune receptor chains. The schema and file format for **Rearrangements** has been previously described (Vander Heiden et al., 2018) and are included in the AIRR Data Model with minor enhancements to support linking back to the **Repertoire**.

### Relationships Between MiAIRR Objects

The MiAIRR objects in the six high-level sets are hierarchical, and include information about the study, the subjects, the collected samples and how they are processed, details of the sequencing protocol, and information about the data analyses. Standard terminology is used to describe the relationships between the schema objects. The top-down relationships are either 1-to-n indicating the single top-level object can be related to any number of sub-level objects, or n-to-n indicating any number of top-level objects can be related to any number of sub-level objects. Lastly, 1-to-1 indicates the single top-level object is related to a single sub-level object.

- Study 1-to-n with Subject. A study may contain any number of subjects.
- Subject 1-to-n with Diagnosis. Each subject may have any number of diagnoses.
- Subject 1-to-n with Sample. Each subject may be associated any number of samples.
- Sample 1-to-n with CellProcessing. A sample may have any number of cell processing records.
- CellProcessing 1-to-n with NucleicAcidProcessing. A cell processing record may have any number of nucleic acid processing records.
- NucleicAcidProcessing 1-to-n with SequencingRun. A nucleic acid processing record may have any number of sequencing runs.
- SequencingRun n-to-n with DataProcessing. Multiple sequencing runs can be combined in a data processing step, and multiple data processing actions can be performed on a sequencing run.

### Repertoire Schema

To simplify the processing of the multi-layered MiAIRR hierarchy, it has been denormalized around the conceptual *Repertoire* object. A *Repertoire* is an abstract organizational unit of analysis that is defined by the researcher and consists of study metadata, subject metadata, sample metadata, cell processing metadata, nucleic acid processing metadata, sequencing run metadata, a set of raw sequence files, data processing metadata, and a set of rearrangements. A *Repertoire* gathers all of this information together into a composite object, which can be easily accessed by computer programs for data entry, analysis, and visualization. A *Repertoire* is specific to a single subject but can consist of any number of samples (which can be processed in different ways), any number of raw sequence files, and any number of rearrangements. It can also consist of any number of data processing metadata objects that describe the processing of raw sequence files into *Rearrangements*.

A *Repertoire* object corresponds to the biological concept of an immune repertoire sample, a portion of an immune repertoire collected from a single subject and which the researcher experimentally measures and computationally analyzes. However, different researchers can be interested in different subsets of immune repertoire samples; therefore, the schema attempts to be flexible and broadly useful for all AIRR-seq studies. In particular, another researcher can take the same raw sequencing data and associated metadata and create their own *Repertoire* that is different from the original researcher's. For example, the original researcher may analyze all productive Ig heavy chain rearrangements in a sample, while a new researcher may take the study data and focus on the subset of sequences corresponding to rearrangements using the V genes from the IGHV4 family. This new *Repertoire* would have much of the same metadata as the original *Repertoire*, except associated with a different study, and with additional information in the data processing metadata that describes how the rearrangements were filtered down to just the “productive rearrangements for IGHV4.” Likewise, another researcher may get access to the original biosample material and perform their own sample processing and sequencing, which also would be a new *Repertoire*. That new *Repertoire* could combine samples from the original researcher's *Repertoire* with the new sample data to create a larger dataset for the subject.

**Figure 2:**
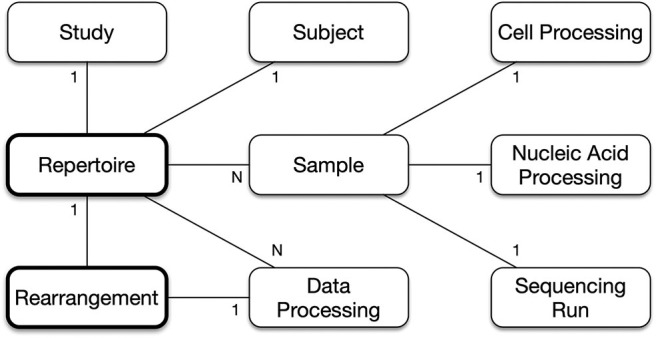
Repertoire and Rearrangement Relationships. A Repertoire object is linked to a single study, a single subject, any number of samples where each sample combines together the cell processing, nucleic acid processing and sequencing run information, and any number of data processing analyses. A Rearrangement object is linked to a single Repertoire object and to a single data processing analysis object.

The denormalization of the hierarchy into a composite *Repertoire* object represents many relationships as 1-to-1, which simplifies the structure as shown in Figure 2. A single *Repertoire* has these relationships with the primary schema objects.

- Repertoire 1-to-1 with Study. A *Repertoire* is for a single study, and a study may have multiple *Repertoire* objects.
- Repertoire 1-to-1 with Subject. A *Repertoire* is for a single subject, though a subject may have other *Repertoire* objects.
- Repertoire 1-to-n with Sample. Generally, a *Repertoire* has a single sample, but sometimes studies perform technical replicates or re-sequencing to generate additional data, and these studies will have multiple samples, which are to be combined and analyzed together as part of the same *Repertoire*.
- Sample 1-to-1 with CellProcessing, NucleicAcidProcessing, and SequencingRun. A sample is associated with a single chain of *SampleProcessing* from initial collection, through cell and nucleic acid processing, to sequencing.
- Repertoire 1-to-n with DataProcessing. A *Repertoire* can be analyzed multiple times.
- Repertoire 1-to-n with Rearrangements. Every single rearrangement is associated with a single *Repertoire*. Even if the exact same rearrangement, in terms of sequence identity and annotation, is found in two or more subjects (e.g., public T-cell receptors), those rearrangements are considered separate objects and have separate links to their *Repertoire* object.

The trade-off with denormalization of the hierarchy is that it causes duplication of data. For example, two *Repertoire* objects for the same study will have the *Study* information duplicated within each of the two *Repertoire* records; likewise, multiple *Repertoire* objects for the same subject will have the *Subject* information duplicated. This trade-off is justified by increased computational efficiency for analysis tools, as it allows quick access to all repertoire metadata without following links and requiring multiple lookups.

While the denormalized repertoire simplifies read-only access to the MiAIRR information, it complicates data entry and write access to the information, because updates need to be propagated to all of the duplicate records. Therefore, *Repertoire* was designed to be easily transformed into a normalized form, representing the full hierarchy of the objects, by utilizing the *study_id, subject_id*, and *sample_id* fields to uniquely identify the Study, Subject, and Sample objects across multiple *Repertoire* objects. The exception is that *CellProcessing* and *NucleicAcidProcessing* do not have their own unique identifiers, as they are included within *Sample*, and *sample_processing_id* can be used to uniquely identify that combination of objects.

### Multiple Data Processing on a Repertoire

Data processing can be a complicated, multi-stage process. Documenting the process in a formal way is challenging, because of the diversity of actions that may be performed. The MiAIRR standard requires documentation of the process but only as free text without any formalized or machine-readable structure. A repertoire might undergo multiple different data processing approaches for any number of reasons, e.g., to compare the results from different toolchains, or to compare different settings for the same toolchain.

It is expected that all of the *Samples* of a *Repertoire* will be processed together within a *DataProcessing* object. That is, data processing that only uses some but not all samples in a *Repertoire* could be confusing to users and appear as though data is missing. Likewise, processing some samples within a repertoire with one data processing approach and the remaining samples with a different data processing approach could also confuse users. Because *DataProcessing* contains unstructured information, it is not possible to validate that all *Samples* in a *Repertoire* are being processed together, so this expectation cannot be strictly enforced.

Having multiple *DataProcessing* objects for a single *Repertoire* object will create multiple sets of *Rearrangements* that are distinct and separate from each other. Analysis tools need to be careful not to mix these sets of rearrangements from different *DataProcessing* objects because it can generate incorrect results. The identifier *data_processing_id* was added so *Rearrangements* can identify their specific *DataProcessing* object.

### AIRR Extension Properties

The OpenAPI v2.0 specification provides the ability to define extension properties on schema objects. These are additional properties on the schema definition directly, not to be confused with additional properties on the data. These extension properties allow those schema definitions to be annotated with MiAIRR and AIRR specific information. Instead of creating separate extensions for each property, a single *x-airr* extension is defined, which is an object that contains any number of properties. Within the AIRR schema, *AIRR_Extension* defines the schema for the *x-airr* object and the properties within it. Table 2 provides a list of the currently supported AIRR extension properties.

**Table 2:** AIRR extension properties.

**Extension**	**Description**
miairr	MiAIRR requirement level.
set	The MiAIRR set for the annotated property.
subset	The MiAIRR subset for the annotated property.
format	Describes the format for the annotated property. Value is either free text, controlled vocabulary or ontology.
ontology	If format=ontology then this provides additional information about the ontology including draft status, name, URL and top node term.
nullable	True if the field can have a null value, otherwise False. Default is True.
identifier	True if the field is an identifier required to link metadata object in the AIRR Data Model. Default is False.
adc-query-support	True if an ADC API implementation must support queries on the field. If False, query support for the field is optional. Default is False.
deprecated	True if the field has been deprecated from the AIRR Schema. Default is False.
deprecated-description	Information regarding the deprecation of the field.
deprecated-replaced-by	The deprecated field is replaced by this list of fields.

### Duality Between Repertoires and Rearrangements

There is an important duality relationship between *Repertoire* and *Rearrangements* objects, specifically between the experimental protocols described in the *Repertoire* object and the annotations on sequences in the *Rearrangements* object. A *Repertoire* defines an experimental design for what a researcher intended to measure or observe, while the analyzed sequences in *Rearrangements* are what was actually measured and observed. Technically, the border between the two occurs at sequencing, that is when the biological physical entity (prepared DNA) is measured and recorded as information (nucleotide sequence). A description of the data processing (which operates on the nucleotide sequence and rearrangement annotations) is included in *Repertoire* for convenience and simplicity, but also to reinforce the presumption that the data processing operates on all of the samples for that *Repertoire*.

This duality is important when considering how to answer certain questions. For example, the *locus* field for rearrangements in *Rearrangements* may have the value “IGH,” which indicates that B cell heavy chain sequences were annotated, yet *Repertoire* might have “T cell” in the *sample.cell_subset* field, which indicates the researcher intended to sequence T cell receptors. This conflict between the two would indicate that something is wrong. Differences can occur in many ways, as with errors in the experimental protocol, or data processing might have incorrectly processed the raw sequencing data leading to invalid annotations.

### Linking Data

An AIRR dataset, stored in files, is always composed of at least two files. One file containing repertoire metadata and another file containing rearrangement data. The rearrangement data is assumed to be significantly larger in size than the repertoire metadata, and thus they are kept in separate files, with rearrangements having their own schema and file format (Vander Heiden et al., 2018). When repertoires and rearrangements are stored in data repositories, the file distinction disappears. Instead, separate query endpoints serve to distinguish between the two.

Regardless of the data storage, identifiers are utilized to link the rearrangement data with its appropriate *Repertoire* object. In particular, each *Repertoire* has a unique *repertoire_id* identifier. This identifier should be globally unique so that *Repertoire* from multiple studies can be combined together without conflict. A simple technique for data repositories to implement global uniqueness is combining a unique identifier string for the repository (e.g., “my-repository.org”) with an integer that is unique within the repository. The *repertoire_id* is used to link other AIRR data to a *Repertoire*. Specifically, the *Rearrangements* schema includes *repertoire_id* for referencing the specific *Repertoire* for that set of rearrangements.

If a *Repertoire* has multiple *DataProcessing* objects, then the *data_processing_id* identifier field should be used to distinguish the various *DataProcessing* objects within the *Repertoire*. The *Rearrangements* schema contains *data_processing_id* for this purpose. The *data_processing_id* is only unique within a *Repertoire*, so *repertoire_id* should first be used to get the appropriate *Repertoire* object and then *data_processing_id* used to acquire the appropriate *DataProcessing* object. It is expected that a typical *Repertoire* will only have a single data processing object, in which case *repertoire_id* and *data_processing_id* will be semantically equivalent and only the former needs to be used.

If a *Repertoire* has multiple samples, then the *sample_processing_id* identifier field should be used to distinguish the various *SampleProcessing* objects within the *Repertoire*. The *SampleProcessing* object uniquely identifies a specific combination of *Sample, CellProcessing, NucleicAcidProcessing*, and *SequencingRun* metadata for the *Repertoire*. A *Rearrangement* object can contain a *sample_processing_id* to identify the *SampleProcessing* object within a Repertoire. The *sample_processing_id* is only unique within a *Repertoire*, so *repertoire_id* should first be used to get the appropriate *Repertoire* object and then *sample_processing_id* used to acquire the appropriate *SampleProcessing* object.

### File Format Specification

Repertoire metadata stored in files should be in YAML/JSON format with a structure defined below. Files should be encoded as UTF-8. Identifiers are case-sensitive. Files should have an extension of yaml, yml, or json.

- The file as a whole is considered a dictionary (key/value pair) structure with the keys *Info* and *Repertoire*.
- The file can (optionally) contain an *Info* object, at the beginning of the file, based upon the *Info* schema in the OpenAPI V2 specification. If provided, the version in *Info* should reference the version of the AIRR Schema for the file.
- The file should correspond to a list of *Repertoire* objects, using *Repertoire* as the key to the list.
- Each *Repertoire* object should contain a top-level key/value pair for *repertoire_id* that uniquely identifies the *Repertoire*.
- The structure is the same regardless of whether the data is stored in a file or queried from a data repository. For example, the ADC API described below will return a properly structured JSON object that can be saved to a file and used directly without modification.

## AIRR Data Commons (ADC) API

The AIRR Data Commons (ADC) API provides programmatic access to query and download AIRR-seq data. The ADC API is a web API that uses JSON as its communication format and standard HTTP methods of GET and POST. The ADC API is read-only, and the mechanism of inclusion of AIRR-seq datasets into a data repository is left up to the repository. Furthermore, repositories are free to choose the internal data representation and storage for their databases, but, to be considered an AIRR-compliant Data Repository as part of the AIRR Data Commons, AIRR-seq data returned from the ADC API must conform to the structure and format according to the AIRR Data Model. The design of the ADC API was greatly inspired by National Cancer Institute's Genomic Data Commons (GDC) API (Jensen et al., 2017). The ADC API is provided in a machine-readable YAML document that follows the OpenAPI v2.0 specification, and this paper describes Version 1 of the ADC API. The following sections describe in detail the API endpoints and the components of an API request.

### ADC API Endpoints

The ADC API is versioned with the version number (v1) as part of the base path for all endpoints. Each ADC API endpoint represents specific functionality as summarized in Table 3. The ADC API has two classes of endpoints. The endpoints that respond to GET requests are simple services that require few or no parameters. While the endpoints that respond to POST requests are the main query services and provide many parameters for specifying the query as well as the data in the API response.

**Table 3:** ADC API endpoints.

**Endpoint**	**Type**	**HTTP method**	**Description**
/v1	Service status	GET	Returns success if API service is running.
/v1/info	Service information	GET	Upon success, returns service information such as name, version, etc.
/v1/repertoire/: repertoire_id	Retrieve a repertoire given its repertoire_id	GET	Upon success, returns the Repertoire information in JSON according to the AIRR Data Model.
/v1/repertoire	Query repertoires	POST	Upon success, returns a list of Repertoires in JSON according to the AIRR Data Model.
/v1/rearrangement/: sequence_id	Retrieve a rearrangement given its sequence_id	GET	Upon success, returns the Rearrangement information in JSON or TSV format according to the AIRR Data Model.
/v1/rearrangement	Query rearrangements	POST	Upon success, returns a list of Rearrangements in JSON or TSV format according to the AIRR Data Model.

The ADC API v1 provides two primary endpoints for querying and retrieving AIRR-seq data. The *repertoire* endpoint allows querying upon any field in the *Repertoire* schema including study, subject, sample, cell processing, nucleic acid processing, sequencing run, raw sequencing files, and data processing information. Queries on the content of raw sequencing files are not supported, but queries on file attributes, such as name, type, and read information, are supported. Queries on rearrangements are provided by the *rearrangement* endpoint.

The standard workflow to retrieve all of the data for an AIRR-seq study involves performing a query on the *repertoire* endpoint to retrieve the repertoires in the study, and one or more queries on the *rearrangement* endpoint to download the rearrangement data for each repertoire. The endpoints are designed so the API response can be saved directly into a file and be used by AIRR analysis tools, including the AIRR Python and R reference libraries, without requiring modifications or transformation of the data.

#### Repertoire Endpoint

The *repertoire* endpoint provides access to all fields in the *Repertoire* schema. There are two types of endpoints; one for retrieving a single repertoire given its identifier, and another for performing a query across all repertoires in the data repository.

It is expected that the number of repertoires in a data repository will never become so large that queries become computationally expensive. A data repository might have thousands of repertoires across hundreds of studies, yet such numbers are easily handled by databases. Based upon this, the ADC API does not place limits on the *repertoire* endpoint for the fields that can be queried or the operators that can be used.

#### Rearrangement Endpoint

The *rearrangement* endpoint provides access to all fields in the *Rearrangements* schema. There are two type of endpoints; one for retrieving a single rearrangement given its identifier, and another for performing a query across all rearrangements in the data repository.

Unlike repertoire data, data repositories are expected to store millions or billions of rearrangement records, where performing “simple” queries can quickly become computationally expensive. Data repositories are encouraged to optimize their databases for performance. Therefore, based upon a set of query use cases provided by immunology experts, a minimal set of required fields was defined that can be queried. These required fields are described in Table 4.

**Table 4:** Required queryable fields for rearrangements.

**Field(s)**	**Description**
sequence_id, repertoire_id, data_processing_id, sample_processing_id, clone_id, cell_id	Identifiers; *sequence_id* allows for query of that specific rearrangement object in the repository, while *repertoire_id, data_processing_id*, and *sample_processing_id* are links to the repertoire metadata for the rearrangement. The *clone_id*, and *cell_id* identifiers allow for the grouping of rearrangements based on clone assignment and single cell assignment.
locus, v_call, d_call, j_call, c_call, productive, junction_aa, junction_aa_length	Commonly used rearrangement annotations.

### Components of an API Request

The ADC API specifies the query parameters in Table 5. These are only applicable to the repertoire and rearrangement query endpoints, i.e., the HTTP POST endpoints. Fields are specified using the full traversal path from the top-level object down to the specific field using the dot (“.”) as the separator. Currently this is only applicable for repertoire queries as the rearrangement schema does not have any nested objects. Examples for repertoire queries include study title (“study.study_title”), tissue (“sample.tissue”), cell subset (“sample.cell_subset”), organism taxonomic identifier (“subject.organism.id”), and data processing identifier (“data_processing.data_processing_id”).

**Table 5:** Query parameters for ADC API.

**Parameter**	**Default**	**Description**
filters	Null	Specifies logical expression for query criteria.
format	JSON	Specifies the API response format: JSON, AIRR TSV.
fields	Null	Specifies which fields to include in the response.
include_fields	Null	Specifies set of AIRR Standard fields to be included in the response. Options are “miairr” for only the MiAIRR fields, “airr-core” for the MiAIRR, AIRR required and identifier fields, and “airr-schema” for all fields in the AIRR Schema.
from	0	Specifies the first record to return from a set of search results.
size	Repository dependent	Specifies the number of results to return.
facets	Null	Provide aggregate count information for the specified field.

#### Filters Query Parameter

The *filters* parameter enables passing complex query criteria to the ADC API. The parameter represents the query in a JSON object. A *filters* query consists of an operator (or a nested set of operators) with a set of field and value operands. The query criteria as represented in a JSON object can be considered an expression tree data structure where internal nodes are operators and child nodes are operands. The expression tree can be of any depth, and recursive algorithms are typically used for tree traversal in processing the parameter. The operators specified by the ADC API are given in Table 6.

**Table 6:** Query operators for ADC API.

**Operator**	**Operands**	**Value data types**	**Description**	**Example**
=	Field and value	String, number, integer, or boolean	Equals	junction_aa = “CASSYIKLN”
!=	Field and value	String, number, integer, or boolean	Does not equal	subject.organism.id != 9606
<	Field and value	Number, integer	Less than	sample.cell_number <1000
< =	Field and value	Number, integer	Less than or equal	sample.cell_number < = 1000
>	Field and value	Number, integer	Greater than	sample.cell_number > 10000
>=	Field and value	Number, integer	Greater than or equal	sample.cell_number >= 10000
isis missing	Field	n/a	Is missing	sample.tissue is missing
notis not missing	Field	n/a	Is not missing^*^	sample.tissue is not missing
in	Field, multiple values in a list	String, number, or integer	Matches a string or number in a list	subject.strain_name in [“C57BL/6”, “BALB/c”, “NOD”]
exclude	Field, multiple values in a list	String, number, or integer	Does not match any string or number in a list	subject.strain_name exclude [“SCID”, “NOD”]
contains	Field and value	String	Contains the substring	study.study_title contains “cancer”
and	Multiple operators	n/a	Logical AND	(subject.organism.id != 9606) and (sample.cells_per_reaction >= 10000) and (subject.strain_name exclude [“SCID”, “NOD”])
or	Multiple operators	n/a	Logical OR	(sample.cell_number <1000) or (sample.tissue is missing) or (subject.organism.id exclude [9606, 10090])

* *Note that the not operator is different from a logical NOT operator, and the logical NOT is not required as the other operators provide negation*.

#### Format Query Parameter

The *format* parameter specifies the format of the data in the API response. JSON is the default format and the only format available for all endpoints except for the *rearrangement* endpoint which accepts “tsv” for the AIRR TSV format.

#### Fields Query Parameter

The *fields* parameter specifies which fields are to be included in the API response. By default, all fields stored in the data repository are returned in the API response.

#### Include Fields Query Parameter

The *include_fields* parameter specifies that the API response should include a well-defined set of AIRR Standards fields. This parameter can be used in combination with the *fields* parameter to restrict or expand the fields returned in the response. This is a convenience parameter to ensure that specific AIRR fields are returned without requiring those fields to be individually provided with the *fields* parameter. Any fields that lack a value will be assigned *null* in the response.

#### From and Size Query Parameters

The ADC API provides a pagination feature that limits the number of results returned by the API. The *from* query parameter specifies which record to start from when returning results. This allows records to be skipped. The default value is 0 indicating that the first record in the set of results will be returned.

The *size* query parameter specifies the maximum number of results to return in a single web API request. Additional results can be retrieved by performing additional web API requests. The default value is specific to the data repository, and a maximum value may be imposed by the data repository. This is to prevent queries from “accidentally” returning millions of records. The *info* endpoint provides the data repository default and maximum values for the *repertoire* and *rearrangement* endpoints, which may have different values. A value of 0 indicates there is no limit on the number of results to return, but if the data repository does not support this, then the default value will be used.

The combination of *from* and *size* can be used to implement pagination in a graphical user interface, or to split a very large download into smaller batches. For example, if an interface displays 10 records at a time, the request would assign *size*=*10* and *from*=*0* to get the ten results to display on the first page. When the user traverses to the “next page,” the request would assign *from*=*10* to skip the first ten results and return the next ten results, and *from*=*20* for the next page after that, and so on.

#### Facets Query Parameter

The *facets* parameter aggregates count information for the specified field. Currently, only a single field can be specified. It provides all values that exist for the field, and the number of records (repertoires or rearrangement) that have this value. The *facets* parameter can be used in conjunction with the *filters* parameter to get aggregate information for a set of search results. Common use of this functionality might be to display aggregate information in a graphical user interface or to generate simple visualizations of single field statistics (e.g., a histogram of CDR3 lengths).

## AIRR Data Commons API Implementations

### Previous API Implementations

As a steppingstone to the AIRR Community endorsed ADC API, and in parallel with the early development of the MiAIRR and AIRR Data standards, several groups from within the AIRR Community established a preliminary query API for AIRR-seq repositories. Designed to work with the iReceptor Scientific Gateway (Corrie et al., 2018), the iReceptor API provided a set of query mechanisms against AIRR-seq repositories, with an ability to filter both repertoire and rearrangement data in those repositories. The iReceptor API was implemented against two AIRR compatible repositories, the iReceptor Public Archive (Corrie et al., 2018) and the VDJServer repository (Christley et al., 2018). This API was used in the iReceptor Scientific Gateway to query and federate data across these distributed repositories (Corrie et al., 2018). With the introduction of the ADC API, the iReceptor API will be deprecated and the iReceptor Scientific Gateway, the iReceptor Public Archive repository, and the VDJServer repository have implemented the ADC API as their primary web API interface for the AIRR Data Commons.

### Reference Implementation for ADC API Service

The AIRR Community provides a reference implementation for an ADC API service. The reference implementation can be utilized for any number of tasks. For example, a data repository might use the source code as a starting point for their own implementation and can compare the behavior of their service against the reference. Another example is a tool developer, who wishes to use the API, can setup a local data repository so they can develop and test their tool before sending API requests across the internet to remote data repositories. While the reference implementation is functionally complete, it has minimal security and no optimizations for large data, so it should not be used directly for production systems.

The reference implementation consists of four GitHub repositories:

- adc-api, top-level service composition;
- adc-api-js-mongodb, a JavaScript web service that responds to API requests and queries a MongoDB database;
- adc-api-mongodb-repository, a MongoDB database for holding AIRR-seq data;
- adc-api-tests, a suite of functional tests and a test dataset.

Docker and docker-compose are used to provide a consistent deployment environment and compose the multiple components together into a single service. Complete documentation for configuring and deploying the reference implementation is available in the adc-api repository as well as the AIRR Community documentation website (https://docs.airr-community.org).

### The iReceptor ADC API Implementation

The iReceptor Project (Corrie et al., 2018) operates a cluster of AIRR compliant repositories as nodes in the AIRR Data Commons. An implementation of the ADC API to query these repositories has been developed. The iReceptor Project also operates the iReceptor Scientific Gateway (https://gateway.ireceptor.org), a web portal that uses the ADC API to find, access, and search repositories in the AIRR Data Commons. Finally, the iReceptor Turnkey is an AIRR compliant repository that is easy to download and install, providing a mechanism for research groups to operate their own repository as a node in the AIRR Data Commons. The iReceptor Turnkey uses Docker to compose a MongoDB database, a web API query service implementation, and data loading and performance monitoring containers. The iReceptor Turnkey provides a full, optimized implementation of the ADC API and is available for download on GitHub (https://github.com/sfu-ireceptor/turnkey-service-php).

### The VDJServer ADC API Implementation

The VDJServer analysis portal (Christley et al., 2018) allows researchers to analyse their AIRR-seq data and to publish and publicly share their study data, metadata, analyses, and results through the VDJServer Community Data Portal (CDP). Data in the VDJServer CDP can be queried and downloaded through the ADC API. The VDJServer implementation of the ADC API consists of a JavaScript API service which communicates with a scalable, high-performance database managed by the Texas Advanced Computing Center. While currently restricted to publicly published studies, the VDJServer ADC API is integrated with VDJServer's user authentication mechanisms and analysis capabilities, so a future release of VDJServer will allow users to query their private data through the ADC API as well as directly run analysis jobs on queried data.

## Example Use Case

We provide an example use case to demonstrate how to query the ADC API, save the downloaded repertoire and rearrangement data into AIRR standard formatted files, and perform an analysis on the data. The example uses the AIRR standards python library and docker image. A walkthrough is provided in Box 1 as well as on the AIRR Community documentation website (https://docs.airr-community.org). The example is split between two python scripts; one that performs the query and saves the data into files, and another that reads the data from the files and generates a grouped CDR3 amino acid length distribution plot. The two scripts could be combined into one, but this example illustrates how the queried data can be saved into files for later use. The example uses the AIRR standards python library.

Box 1 **Walkthrough**

We will use the airr-standards docker image for this example, which comes loaded with all the python packages needed. You will want to map a local directory inside the docker container so you can access the data and analysis results afterwards:

# Download the image

docker pull airrc/airr-standards:latest

# Make local temporary directory to hold the data

mkdir adc_example

cd adc_example

# Invoke a shell session inside the docker image

docker run -it -v.:/data airrc/airr-standards:latest bash

The first python script queries the data from the VDJServer data repository and saves them into files:

*# Query the data*

cd /data

python3/airr-standards/docs/examples/api/retrieve_data.py

Only a subset of the data is downloaded for illustration purposes. Review the code to see how all data can be downloaded. A total of 40 repertoires and 293,414 rearrangements should be downloaded. The repertoire metadata is saved in the *repertoires.airr.json* file, and the rearrangements are saved in the *rearrangements.tsv* file. The script should take a few minuteminutesminute to run and produce the following display messages:

       Info: AIRR Data Commons API

      version: 1.3

description: API response for repertoire query

Received 40 repertoires.

Retrieving rearrangements for repertoire: 2366080924918616551-242ac11c-0001-012

Retrieved 10000 rearrangements for repertoire: 2366080924918616551-242ac11c-0001-012

Retrieving rearrangements for repertoire: 2541616238306136551-242ac11c-0001-012

Retrieved 6114 rearrangements for repertoire: 2541616238306136551-242ac11c-0001-012

Retrieving rearrangements for repertoire: 1993707260355416551-242ac11c-0001-012

Retrieved 10000 rearrangements for repertoire: 1993707260355416551-242ac11c-0001-012

Retrieving rearrangements for repertoire: 2197374609531736551-242ac11c-0001-012

Retrieved 10000 rearrangements for repertoire: 2197374609531736551-242ac11c-0001-012

[remaining output snipped for brevity…]

The seconds python script loads the data from the files and generates a CDR3 amino acid length distribution that is grouped by the T cell subset. This study performs flow sorting to generate four T cell subsets: naive CD4+, naive CD8+, memory CD4+, memory CD8+. The script uses the repertoire metadata to determine the T cell subset for the rearrangement, tabulates the counts, normalizes them, and generates a grouped bar chart with the results:

# *Run the analysis*

python3 /airr-standards/docs/examples/api/analyze_data.py

**Figure 3:**
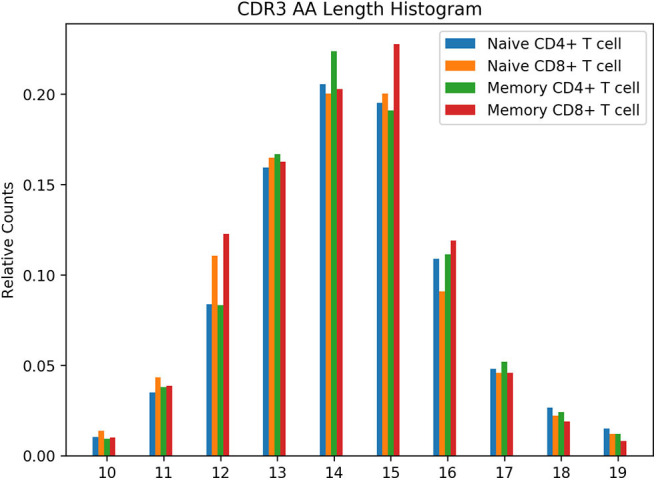
CDR3 AA Length Histogram from Example Use Case Walkthrough. Normalized count for CDR3 lengths from 10 to 19 amino acids is shown for four T cell subsets. As only partial data was downloaded in the example for illustrative purposes, this figure should not be construed as representing the true length distribution of the repertoires.

The figure is placed in the *plot.png* file and should look like Figure 3.

### Study Data

This example retrieves data for a single study (Rubelt et al., 2016) identified by its MiAIRR study ID, which is identified by NCBI BioProject accession PRJNA300878. Some basic information for the study:

- 5 pairs of human twins.
- B cells and T cells sequenced.
- B cells sorted into naive and memory.
- T cells sorted into naive CD4, naive CD8, memory CD4 and memory CD8.
- Total of 60 repertoires: 20 B cell repertoires and 40 T cell repertoires.

In this example, only the T cell repertoires are queried and retrieved. The example shows how the *size* and *from* query parameters can be used to retrieve the data over a series of web requests. Only a subset of the data is downloaded for illustration purposes. The analysis script loads the data from the files and generates a CDR3 amino acid length distribution that is grouped by the T cell subset. It uses the repertoire metadata to determine the T cell subset for the rearrangement, tabulates the counts, normalizes them, and generates a grouped bar chart with the results shown in Figure 3.

## Conclusions

The use of high-throughput sequencing for profiling B-cell and T-cell receptor repertoires has resulted in a rapid increase in data generation. The AIRR Data Commons based upon community standards has established an ecosystem for researchers that makes AIRR-seq data findable, accessible, interoperable, and reusable. This paper describes the AIRR Data Model for repertoires and rearrangements, Version 1 of the ADC API, and standards-based reference implementation tools. As of today, the data repositories accessible through the iReceptor Scientific Gateway allows query of hundreds of repertoires and over two billion rearrangements. This will increase significantly as the ADC API is adopted and new data repositories become available. The accumulation of AIRR-seq data across a wide variety of studies, subjects, diseases, treatments and outcomes will drive insights into the adaptive immune system not achievable with any single study.

### Future Work

The CRWG has already identified a number of potential improvements and enhancements that will be considered for future versions of the ADC API. This is not a complete list but represents some of the main topics to be considered.

- A registry for AIRR compliant repositories. The registry is a discovery mechanism for finding repositories that are AIRR compliant. The registry should be publicly accessible and provide enough information for users of the registry to connect and utilize the repositories.
- More advanced summary statistics. The facets query parameter can provide count information for a single field. A potential enhancement is to allow additional mathematical operations like summation, multiplication, moving averages, etc., that can include multiple fields. Such enhancements would provide analysis capability vs. just query, but databases increasingly have the ability to perform these “aggregation” functions integrated with the query.
- Asynchronous queries. Currently, queries operate in a synchronous fashion. When an API request is made, the connection is blocked until the query finishes and data is returned. As data gets larger, complex queries will take longer to run, which can cause network timeouts and inhibit interactivity. An asynchronous API request would return immediately, vs. blocking, and there would be mechanisms to notify the client when the data is available.
- Query digital object identifiers (DOIs). The ADC API does not require that the service saves the query and give it a unique identifier. However, query DOIs would allow researchers to reference them in journal publications as well as other benefits that DOIs provide. Furthermore, building up a history of queries would allow analysis on the types of queries performed so services can optimize their performance or drive enhancements to the ADC API.
- Data provenance and versioning. The data in a data repository may change over time, and thus the data returned from a query may also change over time. Designs can span the range from keeping a “changelog” document to full data versioning whereby historical queries always return the exact same data. Trade-offs involve storage, performance and complexity considerations against the desire for scientific reproducibility and perfect replication.

As with all AIRR Community working groups, the CRWG is an open community and new members are welcome to join.

## Data Availability Statement

The original contributions presented in the study are included in the article/Supplementary Material.

## Author Contributions

SC led the implementation effort, wrote the manuscript draft, and wrote the ADC API reference implementation. LC, CW, and BC functioned as co-chairs of the working group. All authors researched, discussed, and designed the described standards as part of the AIRR Common Repository Working Group, read, and approved the final manuscript.

## Conflict of Interest

ST was employed by company 10x Genomics. The remaining authors declare that the research was conducted in the absence of any commercial or financial relationships that could be construed as a potential conflict of interest.

## Supplementary Material

Supplementary Material contains the list of endorsing members of the AIRR Community of The Antibody Society.
